# Connections between the Madden–Julian Oscillation and surface temperatures in winter 2018 over eastern North America

**DOI:** 10.1002/asl.869

**Published:** 2018-11-19

**Authors:** Bradford S. Barrett

**Affiliations:** ^1^ Oceanography Department U.S. Naval Academy Annapolis Maryland

**Keywords:** Madden–Julian Oscillation, Rossby wave train, tropical–extratropical teleconnections, surface air temperature variability

## Abstract

From January to March 2018, one of the strongest Madden–Julian Oscillation (MJO) events of the last 45 years progressed eastward along the equator from the Indian Ocean to the Pacific Ocean then back to the Indian Ocean. In response to strong tropospheric heating in the MJO's active convective envelope, several pronounced Rossby wave trains developed and extended from the equatorial tropics, across the extratropical Pacific and North America, and into the extratropical Atlantic. The origins of these Rossby wave trains evolved eastward with time, generally following the eastward progression of the MJO, but preferentially clustered in subtropical India and Southeast Asia and in two locations in the subtropical Pacific Ocean: along 160°E and 170°W. Over eastern North America, surface and lower‐tropospheric temperatures rose to more than 12 °C above normal when the MJO convective envelope was over the Indian Ocean (in mid‐January) and Western Hemisphere (in late February). In between those warm periods, temperatures cooled to below normal while the MJO convection was over the western Pacific. These temperature anomalies evolved in time with the pronounced Rossby wave trains that linked eastern North America with the Tropics in the Eastern Hemisphere: warm temperatures occurred when ridging was present over eastern North America and cooler temperatures occurred when troughing was present. This variability is discussed and placed in context of recent work showing the MJO's role in modulating temperature and circulation.

## 1 INTRODUCTION

Tropical heating in the convectively active region of the Madden–Julian Oscillation (MJO; Madden and Julian, 1971; 1972) has been shown to be an effective source of Rossby waves to the extratropics (Sardeshmukh and Hoskins, 1988; Jin and Hoskins, 1995). To generate Rossby waves, heating associated with the MJO convection leads to a divergent component of the upper‐tropospheric tropical and subtropical flow that advects absolute vorticity poleward and leads to the formation of a wave train (Lin, 2009; Seo and Son, 2012) that can be traced (Seo and Lee, 2017). The subtropical origins of the Rossby wave train seem to depend on the longitude of the convection; namely, the wave train origins can move east, in tandem with the source convection (Johnson and Feldstein, 2010; Hamill and Kiladis, 2014; Stan *et al*., 2017).

MJO‐generated Rossby waves have been shown in both theoretical and observational studies to extend into the extratropics, poleward and eastward away from the convection source (Seo *et al*., 2016). It has been demonstrated that approximately 30% of the total variability of the intra‐seasonal, upper‐level extratropical circulation is associated with MJO‐induced teleconnections (Matthews *et al*., 2004; Seo and Son, 2012; Wang *et al*., 2013). As a result, the MJO has been connected to the modulation of a wide range of weather phenomena. One of the ways in which the MJO's influence extends to North America is via its modulation of the North Atlantic Oscillation (NAO) and Pacific–North American (PNA) pattern. The NAO and PNA are two of the leading modes of extratropical variability in the Northern Hemisphere (Blackmon *et al*., 1984; Hurrell *et al*., 2005). The NAO and MJO have a robust time‐lagged relationship. About 10 days after MJO convection is over the Indian Ocean (IO), the probability of a positive NAO is increased significantly. Similarly, about 10 days after MJO convection is over the western Pacific Ocean (WP), the probability of a negative NAO is increased significantly (Cassou, 2008; Lin *et al*., 2009; Yadav and Straus, 2017). A positive NAO is associated with ridging over the eastern United States and adjacent North Atlantic and warmer‐than‐average surface air temperatures. Conversely, a negative NAO is associated with troughing over the eastern United States and adjacent North Atlantic and colder‐than‐average surface air temperatures Thus, warmer temperatures over the eastern United States can be expected after MJO convection is over the IO, and colder temperature can be expected after MJO convection is over the WP. This relationship between the MJO and NAO appears to be strongest when the El Niño–Southern Oscillation (ENSO) is in negative (La Niña) phase (Roundy *et al*., 2010). The MJO's influence on the extratropics also appears strongest when the quasi‐biennial oscillation (QBO) is in its easterly phase (Yoo and Son, 2016).

Over the Pacific, the PNA pattern has also been found significantly related to the MJO. The MJO's modulation of the PNA pattern is attributed to a negative Rossby wave source north of the enhanced convection over the IO and a positive Rossby wave source north of the enhanced convection over the WP (Seo and Lee, 2017). Indeed, when the MJO convection is east of the Philippines, it tends to excite the positive PNA phase, which translates into positive geopotential height anomalies over western North American, negative anomalies over eastern North America (Higgins and Mo, 1997; Mori and Watanabe, 2008; Franzke *et al*., 2011), and colder‐than‐normal temperatures over the northeastern United States (Leathers *et al*., 1991).

One of the primary consequences of the MJO's connection to the extratropics is its modulation of surface air temperature. Many recent studies agree that warm temperature anomalies are more common over North America when MJO convection is over the Maritime Continent (MC; Lin and Brunet, 2009; Zhou *et al*., 2012; Matsueda and Takaya, 2015), especially when the PNA is negative (Schreck *et al*., 2013). This relationship is due to variability in the Pacific jet and associated wave breaking (Henderson *et al*., 2016), which are connected to a Rossby wave train associated with the MJO tropical convection (Lin *et al*., 2009). This wave train leads to ridging conditions and above‐normal air temperature over the northeastern United States. There is somewhat less agreement as to which MJO phase favors colder temperatures over North America. Zhou *et al*. (2012) noted that cold temperatures over eastern North America occur with greater frequency when the MJO convection is over the IO, but Zhou *et al*. (2011) noted colder temperatures when the convection is over the central and eastern Pacific. Lin and Brunet (2009) found cold temperature anomalies United States–Canada border after MJO convection in the central Pacific Ocean, but Matsueda and Takaya (2015) did not find statistically significant cold anomalies for any phase of the MJO. Snowfall over the northeastern United States is enhanced during phases 8 and 1 (Klotzbach *et al*., 2016), which would be consistent with colder air temperatures there when convection is over the Western Hemisphere. This somewhat mixed signal in the relationship between the MJO and air temperatures in eastern North America provided motivation for this current study. The goal is to analyze variability in lower‐troposphere air temperature in context of a record‐strength, “canonical” eastward propagating MJO event, to help understand the MJO–temperature connection over eastern North America. The remainder of this article is organized as follows: datasets and methodology are presented in section 2; results are presented and discussed in section 3; and conclusions are provided in section 4.

## 2 DATA AND METHODS

The analyses of this study use several publicly available datasets. First, the MJO was characterized using the Real‐time Multivariate MJO (RMM) index (Wheeler and Hendon, 2004). The daily RMM index is based on time series of the first two leading principal components derived from empirical orthogonal functions of equatorially averaged (15°S–15°N) 850‐hPa zonal wind, 200‐hPa zonal wind, and outgoing longwave radiation. The RMM index is divided into eight phases, with each phase corresponding broadly to the geographic location of the MJO enhanced convective signal.

Second, 200‐ and 850‐hPa geopotential heights were taken every 6‐hr from the NCEP‐DOE AMIP‐II Reanalysis from January 1, 2018 to March 14, 2018 at 2.5 × 2.5° grid spacing. Air temperatures at 850 hPa were taken from the NCEP‐DOE reanalysis at the same resolution and for the same period. Daily outgoing longwave radiation (OLR) was taken from the NOAA uninterpolated OLR dataset (https://www.esrl.noaa.gov/psd/data/gridded/data.uninterp_OLR.html), also at 2.5 × 2.5° horizontal grid spacing. Anomalies of both height and temperature were calculated at each grid point from the 1981–2010 long‐term 6‐hr mean for the respective month; daily OLR anomalies were calculated from the daily long‐term mean product (1979–1995) provided by NOAA. Finally, daily snowfall and daily maximum and minimum 2‐m air temperatures and anomalies at Washington, DC (KDCA), Philadelphia (KPHL), New York City (KNYC), and Boston (KBOS) were taken from the daily climate reports issued by the respective local NWS forecast offices from January 1 to March 14. Analyses end when the MJO weakened to below active.

To remove the influence of synoptic‐scale variability, anomalies of 200‐ and 850‐hPa height, OLR, and 850‐hPa temperature were smoothed in time using a 14‐day moving average via the *smooth* function in MATLAB 2017a. No other spatial or temporal smoothing was applied to the data. The ray tracing technique of Seo and Lee (2017) was manually applied to the smoothed daily 200‐hPa height anomalies. The starting location of each wave train was objectively determined as the center point of the height anomaly located closest to the tropical MJO convective region. The remainder of the wave then traced a path through adjacent anomaly centers. Most of the waves originating in India and Southeast Asia terminated at an anomaly center over North America that did not have an adjacent anomaly center of the opposite sign. The rest of the waves originating in India and Southeast Asia, and all of the waves originating over the central Pacific, terminated at an anomaly center east of North America.

## 3 RESULTS

**Figure 1:**
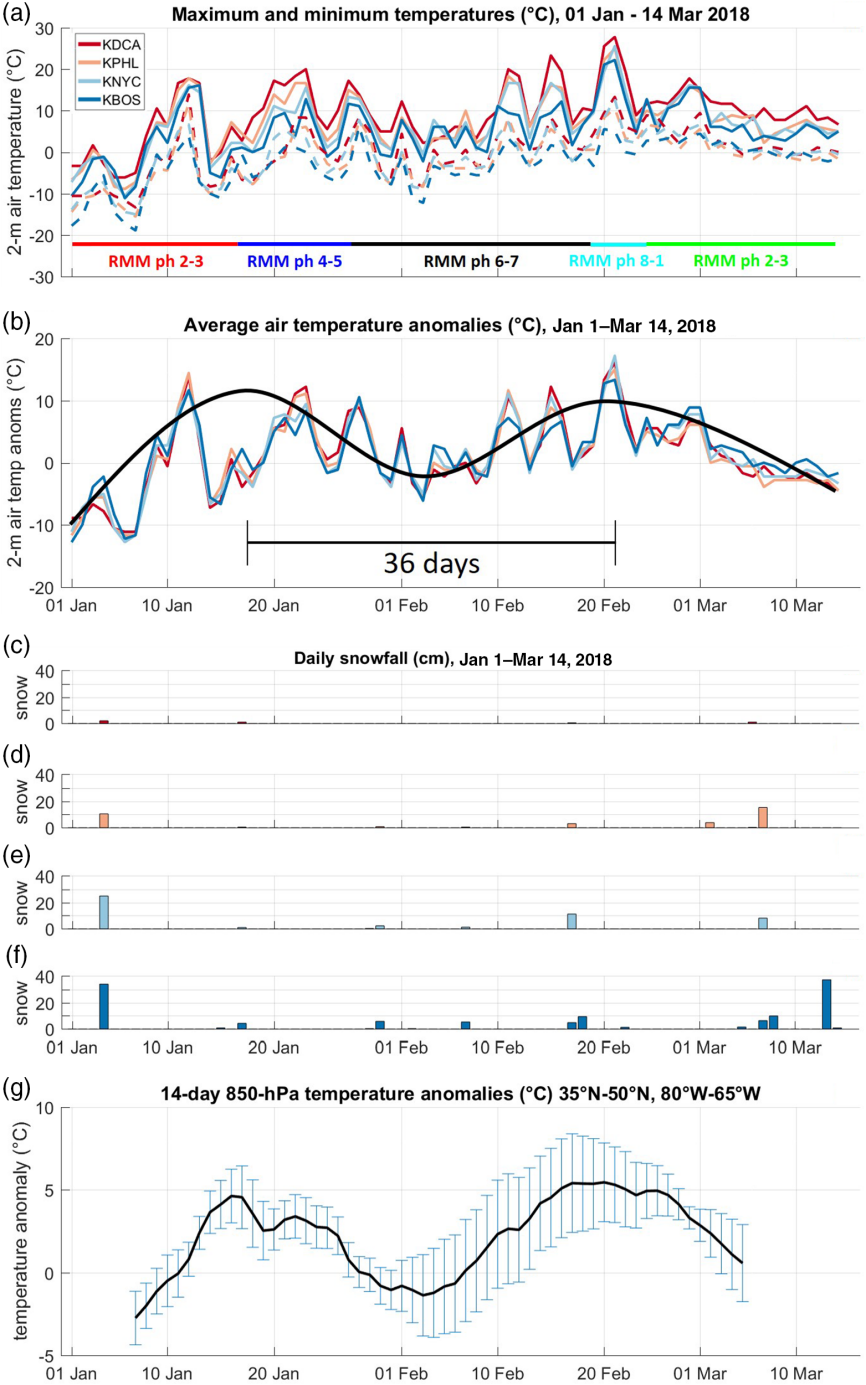
(a) Maximum (solid curves) and minimum (dashed curves) surface air temperatures (in °C) at four stations (Washington, Philadelphia, New York, and Boston) from January 1 to March 14, 2018. Concurrent phase of the MJO indicated by colored horizontal line segments, and color scheme continues for the ray traces in Figures 2a–d and 3a. (b) Anomalies of daily mean temperature (in °C) for the four stations in (a). Anomalies calculated with respect to long‐term daily mean. Fourteen‐day moving average of all four stations shown in dark black curve. Return period (peak‐to‐peak) of 36 days indicated below the temperature curves. (c–f) Daily snowfall amounts at the four stations in (a). (g) Mean (black curve) and standard deviation (blue ranges; *n* = 49 grid points) of anomalous 14‐day smoothed 850‐hPa air temperatures over the northeastern states and adjacent North Atlantic Ocean (35°–50°N, 80°–65°W; region indicated by black box in Figure 2e)

**Figure 2:**
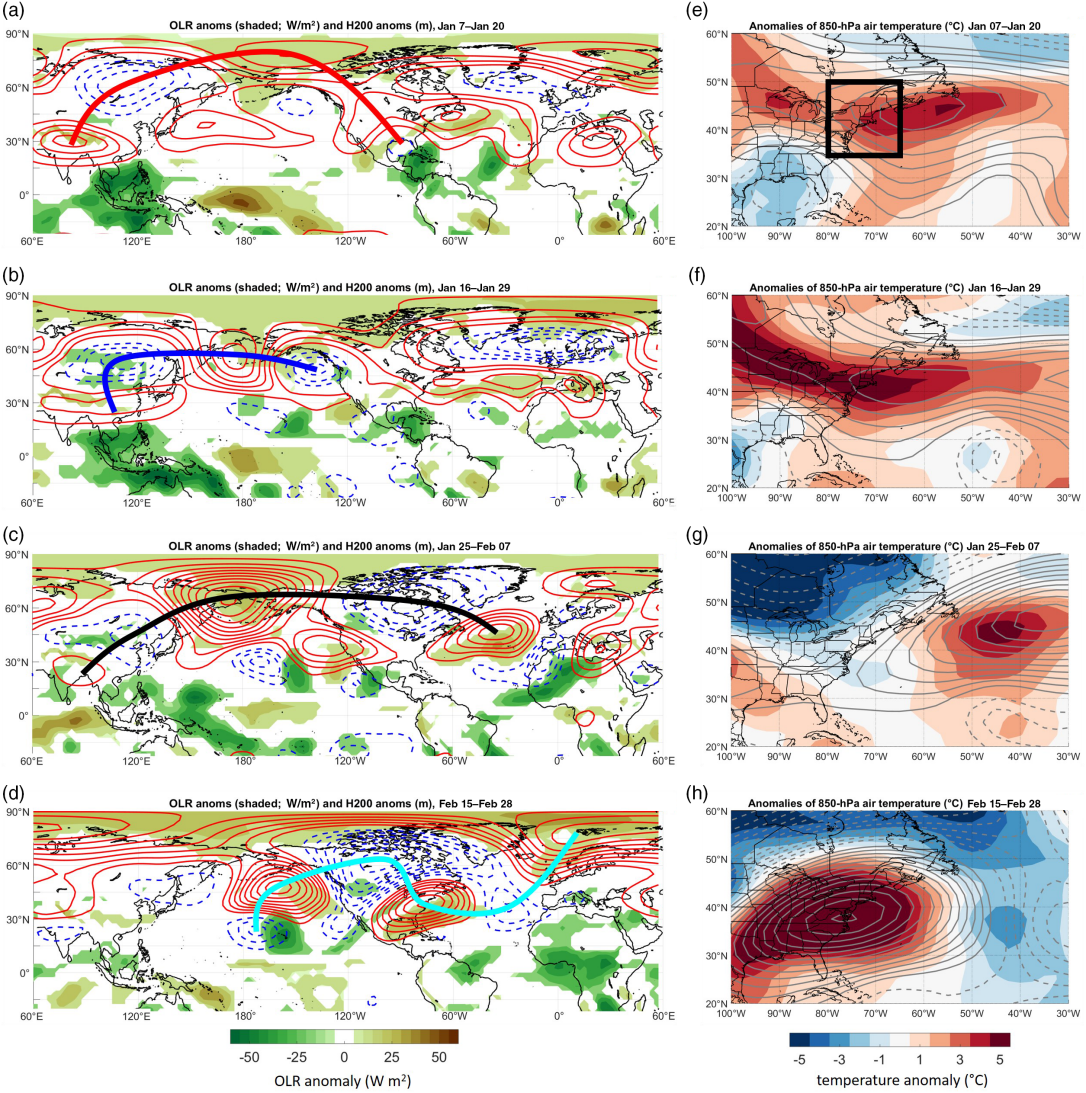
(a–d) Mean 200‐hPa height anomalies (in m; red solid curves show positive anomalies and blue dashed show negative anomalies; contour interval every 45 m) and OLR anomalies (color shading), for (a) January 7–20, 2018; (b) January 16–29, 2018; (c) January 25 to February 7, 2018; and (d) February 15–28, 2018. Rossby wave trains traced in red, blue, black, and cyan curves, respectively, corresponding to MJO phases in Figure 1b. (e–h) Mean 850‐hPa temperature anomalies (in °C) and height (in m; solid contours positive and dashed negative; contour interval 2.5 m) for the same periods in (a–d). Black box in (e) indicates region for spatial averages presented in Figure 1g

From January 1 to March 14, 2018, surface (2 m) air temperatures at four cities along the U.S. east coast ranged from a high of 28 °C (in Washington in mid‐February) to a low of −19 °C (in Boston in January) (Figure 1a). Synoptic‐scale variability on the order of 3–6 days was also evident throughout the period, associated with the passage of frontal systems and mid‐latitude cyclones and anticyclones. Air temperature anomalies at the four cities shifted from near −10 °C in early January to near +13° in mid‐January, then decreased to slightly below normal (−3 °C) in early February (Figure 1b). Temperature anomalies peaked again in late February, exceeding +15 °C, and then declined into early March. The return period between the two peaks in air temperature anomalies was approximately 36 days (Figure 1b), which is strongly suggestive of intra‐seasonal variability. Snowfall in the four cities was mostly confined to the periods of below‐normal temperatures (early January and early March), particularly so for the heavy snow events (days with more than 10 cm) (Figure 1c–f). Mean daily 850‐hPa air temperatures over the northeast region (35–50°N, 80–65°W; shown as a black box in Figure 2e) also featured the same intra‐seasonal pattern of variability (Figure 1g).

**Figure 3:**
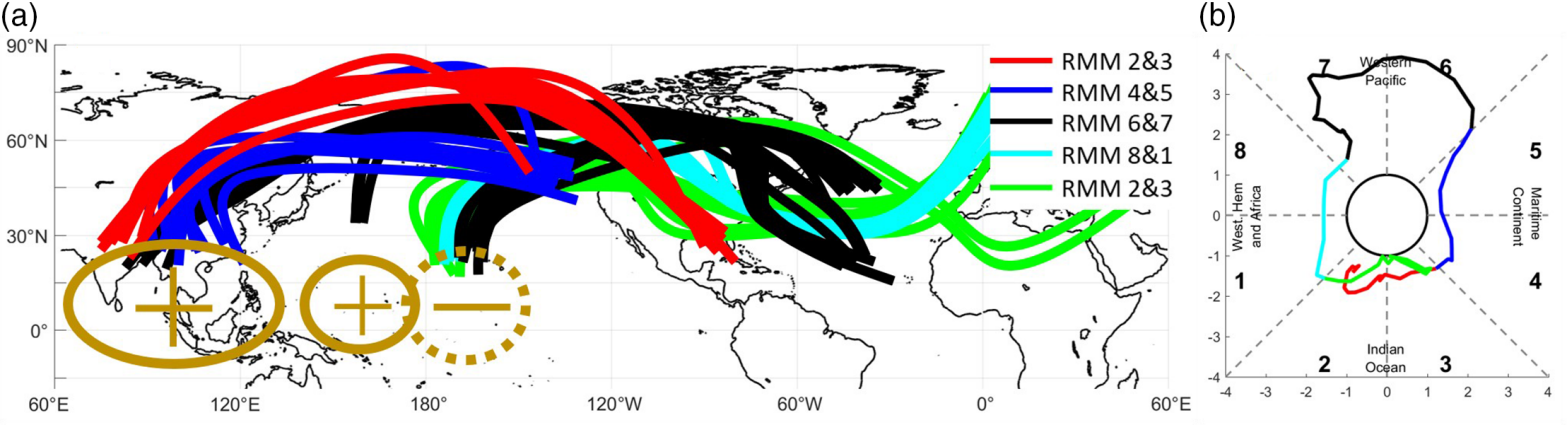
(a) Daily Rossby wave ray traces in 200‐hPa height originating from positive (+ symbol) and negative (− symbol) anomaly centers, from January 1, 2018 to March 14, 2018. Colored lines correspond to MJO phase groupings (red: phases 2 and 3 in January 2018; blue: phases 4 and 5; black: phases 6 and 7; cyan: phases 8 and 1; and green: phases 2 and 3 in March 2018). (b) Daily RMM amplitude and phase from January 1, 2018 to March 14, 2018

During this 2.5‐month period, the MJO completed a full cycle (Figures 1a and 3b). From January 1–16, the MJO convective envelope passed through the IO (phases 2 and 3) with a mean amplitude of +1.7 and a peak of +2.1 (Table 1). The PNA and NAO indices averaged +0.7 and +0.6, respectively, during those days. From January 17–26, the MJO passed through the MC (phases 4 and 5) and was stronger, with a mean amplitude of +1.8 and a peak of +2.5. During that period, the PNA decreased to −0.4 while the NAO remained at about the same intensity (+0.7). The strongest portion of the MJO life cycle occurred from January 27 to February 18, as the MJO transited the WP (phases 6 and 7). In that 21‐day period, the mean MJO amplitude was +3.2, with peak amplitude of +3.93 on February 6. That peak amplitude is the 11th strongest MJO day in the historical record (top 0.07% out of 15,703 days from 1974 to 2018) and the strongest‐ever MJO day for phase 6. A mean MJO amplitude of +3.2 was classified as “extremely active MJO” by LaFleur *et al*. (2015). The PNA during this record‐breaking MJO event was near 0, while the NAO increased to +1.3. From February 19–24, the MJO convective envelope moved across the Western Hemisphere and Africa (phases 8 and 1) with a lower mean (+1.8) and peak (+2.7) amplitude. The PNA amplitude for this period decreased to −0.8, and the NAO amplitude was +0.8. From February 25 to March 14, the MJO weakened to a mean amplitude of +1.4 and peak of +2.2.

**Table 1:** Start and end dates of MJO phase groups, maximum and mean MJO amplitude over those date ranges, mean PNA and NAO index amplitude over the date ranges, and expected and actual surface air temperature anomaly over those date ranges

RMM phases	Start and end dates	Maximum MJO amplitude	Mean MJO amplitude	Mean PNA amplitude	Mean NAO amplitude	Expected surface air temperature anomaly in northeast United States given the…			Actual surface air temperature anomaly
						MJO phase	PNA phase	NAO phase	
2 and 3	Jan 1–Jan 16	2.1	1.7	+0.7	+0.6	− −	− −	++	– – transitioning to ++
4 and 5	Jan 17–Jan 26	2.5	1.8	−0.4	+0.7	++	++	++	++
6 and 7	Jan 27–Feb 18	3.9	3.2	−0.1	+1.3	++	++	++	++ transitioning to − −
8 and 1	Feb 19–Feb 24	2.7	1.8	−0.8	+0.8	− −	++	++	++
2 and 3	Feb 25–Mar 14	2.2	1.4	−0.5	−0.7	− −	++	− −	++ transitioning to − −

One of the consequences of tropical convection, and particularly tropical convection that reaches extreme intensities as measured by the RMM, is the generation of poleward and eastward extended Rossby wave trains. Ray traces of 200‐hPa height anomalies from four active MJO phase groupings are presented in Figure 2a–d. In early–mid January, a positive Rossby wave source was located over India, to the north and northwest of anomalous convection over the eastern IO and MC (Figure 2a). This wave train extended to North America via troughing over Siberia, ridging north of Alaska, troughing in the Gulf of Alaska, and ridging over western Canada (Figure 2a). This wave train was connected to positive temperature anomalies over the New England states and the western Atlantic (Figure 2e). By late January, the wave train had evolved to extend from ridging over Southeast Asia, just north of the tropical convective anomaly over the MC, to troughing over eastern Siberia, to ridging over western Alaska and troughing over the Gulf of Alaska (Figure 2b). This wave train was similar to Straus *et al*. (2015), who also found a Rossby wave source region associated with MJO convection over the MC. This wave pattern coincided with strongly positive temperature anomalies over New England and the western Atlantic (Figure 2f). By early February, MJO convection had shifted into the WP (Figure 2c) with a wave train still extending from Southeast Asia (albeit with weaker 200‐hPa absolute height anomalies, smaller than ±90 m) to a very strong ridge over the Bering Strait (height anomalies of +450 m) to strong troughing over Hudson Bay (height anomalies of −200 m) to ridging again over the North Atlantic (Figure 2c).

This wave pattern coincided with much colder 850‐hPa air temperatures over central and eastern Canada (anomalies below −15 °C), and those cold anomalies edged into the New England states and were associated with below‐normal 850‐hPa heights (−30 m) as well (Figure 2g). By late February, the MJO convection had shifted into the Western Hemisphere (Figure 2d) with a wave train extending from 170°W to a strong ridge over the Gulf of Alaska (height anomalies to +360 m), very strong troughing over western and central Canada (height anomalies to −250 m), and strong ridging over the eastern United States (Figure 2d). At the same time, very strong positive temperature anomalies were located over the entire eastern United States and southeastern Canada (Figure 2h), co‐located with 850‐hPa heights nearly 50 m above normal.

A composite of daily ray traces of wave trains from January 1 to March 14 is shown in Figure 3a. Colorized by RMM phase, the composite shows the eastward progression of the wave train source and roughly follow the evolution of the active convective envelope from the IO (phases 2 and 3) to the MC (phases 4 and 5) to the WP (phases 6 and 7) and Western Hemisphere (phases 8 and 1), then back again to the IO. When MJO convection was in the IO in early–mid January, the wave train source appears to be from a ridge centered over northern India (Figure 3a). As the convection moved east into the MC in late January, the wave train source shifted east as well, to a ridge centered over Southeast Asia. As the convection moved into the WP in early–mid February, the wave train source remained over Southeast Asia before shifting east to the west‐central Pacific (near 160°E) (Figure 3a). The wave source shifted east one more time, to near 170°W, but from there, the waves originated as a trough (instead of as a ridge, as over India and Southeast Asia). As the MJO convection continued to move east through the WP, into the Western Hemisphere (late February), and back into the IO (late February to mid‐March), the negative wave source remained near 170°W (Figures 2d and 3d). The response over eastern North America appeared mostly barotropic, with 200‐ and 850‐hPa height anomalies located over approximately the same geographic regions (Figure 2).

It thus appears that there are several preferred source regions for Rossby wave trains associated with MJO convection: a positive source over India and Southeast Asia, another positive source along 160°E, and a negative source along 170°W (Figure 2d). As the MJO convection progressed eastward, the wave train origins did not exhibit a continuous eastward motion, but rather clustered in space in the three aforementioned regions. Furthermore, the wave trains that began over India and Southeast Asia, and the one over the WP, originated from positive 200‐hPa geopotential height anomalies (see Figure 3a–c). Meanwhile, the wave trains that began over the central Pacific, near 170°W, originated from negative 200‐hPa geopotential height anomalies (see Figure 3d). Nevertheless, regardless of the source location of the convective heating, Rossby wave trains appear to extend significantly eastward and poleward to cover more than half of the Northern Hemisphere.

## 4 CONCLUSIONS

In this study, air temperature variability in winter 2018 over the northeastern Unite States was examined in context of the Madden–Julian Oscillation. In early–mid January 2018, anomalously warm near‐surface air temperatures occurred from Washington to Boston and over the adjacent Atlantic. By early February, temperatures cooled to slightly below‐normal levels, but then warmed again to record levels by mid–late February. The return period between the warm episodes was near 36 days, which is strongly suggestive of intra‐seasonal variability. Concurrent with the temperature variability, a record‐breaking MJO event progressed eastward in the Tropics, from the IO in early January, to the MC and WP in late January and early February, then back to the IO by early March. The amplitude of this MJO event was in the top 0.07% (11 out of 15,703 days), and the highest ever observed in phase 6 (from 1974 to 2018). The severity of the MJO event is also evident from the OLR‐based OMI (Kiladis *et al*., 2014), where the amplitude peaked at 2.83 on February 8, 2018 (a top 1% event). The RMM index responds most strongly to zonal circulation anomalies (Straub, 2013), and the extreme RMM amplitudes in this event (compared to the slightly weaker OMI amplitudes) suggest that this event featured very strong zonal circulations. Those strong circulations then acted as a bridge between the Tropics and extratropics (Henderson *et al*., 2017).

Strong MJO events tend to be preferred during periods of La Niña, since the warmer‐than‐normal sea surface temperatures over the western Pacific warm pool tend to enhance the MJO convection. Stronger MJO events also tend to occur during easterly phases of the QBO, since those phases associate with reduced upper‐tropospheric and lower‐stratospheric stability. Indeed, the January–March 2018 period featured both La Niña and easterly QBO, which acting together, likely not only favored the MJO convection but also strengthened the Rossby wave teleconnections to the extratropics (e.g., Yoo and Son, 2016). Two of the leading modes of extratropical variability, the PNA and NAO, also varied over this period, with the PNA flipping polarity from positive (in early January) to negative (in late January), and the NAO remained positive from January through February before becoming negative in early March.

The evolution of surface air temperatures agrees reasonably well expected evolution according to the polarities of the NAO and PNA (Table 1). The two periods of anomalously warm temperatures occurred while the PNA was negative and the NAO positive, in agreement with previous studies. Furthermore, the minimum in air temperatures between the two warm periods, which occurred in early February, coincided with a 9‐day period when the PNA was negative, also in agreement with previous studies. However, the NAO index not only remained positive during that period of below‐normal temperatures, it continued to strengthen, reaching a peak amplitude on February 9 of +1.8. Normally, a positive NAO index should be associated with above‐normal air temperatures in the northeast states. However, ridging along western North America (which led to a positive PNA index) occurred at the same time as ridging over the central North Atlantic (which led to the positive NAO index). Thus, in between the two ridges, troughing was confined to central North America (Figure 2c), which led to normal to below‐normal temperatures over Canada (Figure 2g) and likely kept temperatures in the northeastern United States from falling too much below normal. These ridges and troughs were connected to wave trains with origins in the Tropics that were directly connected to the extremely active MJO event. Idealized modeling studies that can control both the tropical and extratropical circulation base states are suggested for future work to help understand how extratropical surface temperatures evolve in response to tropical forcing. Future work would also help better understand variability in the origins and path of the Rossby wave response during different phases of ENSO.
